# Preoperative Inflammatory Risk Markers and Postoperative Creatinine and Lactate as Mortality Markers After Surgery for Stanford Type A Acute Aortic Dissection

**DOI:** 10.3390/jcm15197614

**Published:** 2026-10-01

**Authors:** Rauf Önder, Salih Özçobanoğlu

**Affiliations:** 1 Department of Cardiovascular Surgery, Gaziantep Islam Science and Technology University, Gaziantep 27010, Turkey; 2 Department of Cardiovascular Surgery, Gaziantep City Hospital, Gaziantep 27010, Turkey; 3 Department of Cardiovascular Surgery, Antalya University, Antalya 07190, Turkey

**Keywords:** Stanford type A aortic dissection, hemogram-based inflammatory indices, neutrophil-to-lymphocyte ratio, platelet-to-lymphocyte ratio, systemic immune-inflammation index, perioperative mortality, cardiothoracic anesthesia, lactate, creatinine

## Abstract

**Objective:** The primary objective was to evaluate the association between preoperative platelet-to-lymphocyte ratio (PLR), measured at admission, and 30-day mortality after emergency surgical repair for Stanford type A acute aortic dissection (TAAD). Secondary analyses evaluated other preoperative inflammatory and biochemical markers, postoperative biomarker measurements as indicators of evolving postoperative physiology, and their associations with 2-year mortality. **Methods:** This retrospective study included 506 patients who underwent emergency surgery for TAAD. Preoperative and postoperative laboratory parameters—including creatinine, sodium, potassium, lactate, NLR, PLR, SII, and MLR—were collected along with perioperative clinical variables. Group comparisons were performed according to 30-day and 2-year mortality outcomes. Spearman correlation analysis was used to assess associations between biomarkers and clinical parameters. Receiver operating characteristic (ROC) analyses were conducted to determine the discriminative performance of inflammatory indices and biochemical markers. Statistical significance was defined as *p* < 0.05. **Results:** Non-survivors had significantly higher preoperative and postoperative creatinine levels (*p* = 0.008 and *p* = 0.003), longer aortic cross-clamp and circulatory arrest times (both *p* < 0.001), and elevated postoperative lactate levels (*p* = 0.029). All inflammatory indices—including NLR, PLR, and SII—were markedly higher in patients who died within 30 days (all *p* < 0.001). MLR was also significantly elevated in non-survivors, although the magnitude of increase was more modest compared with the other inflammatory indices (preoperative *p* = 0.011; postoperative *p* = 0.007–0.010). In the primary analysis, preoperative PLR showed good discrimination for 30-day mortality (AUC 0.819, 95% CI 0.750–0.889) and remained independently associated with 30-day mortality after adjustment for age, preoperative creatinine, aortic cross-clamp time, and total circulatory arrest time (adjusted OR 1.004, 95% CI 1.002–1.007; *p* < 0.001). Postoperative inflammatory indices, creatinine, and lactate were also associated with mortality and were interpreted as markers of evolving postoperative physiological deterioration, whereas sodium showed no meaningful prognostic contribution. For 2-year mortality, elevated preoperative and postoperative creatinine and lactate levels, together with persistently higher inflammatory indices at all measured time points, were significantly associated with long-term death (all *p* < 0.001). No inflammatory index showed a meaningful correlation with postoperative complications. **Conclusions:** Preoperative PLR was associated with 30-day mortality after TAAD surgery. Postoperative inflammatory indices; creatinine; and lactate were also associated with mortality but may primarily have reflected evolving postoperative physiological deterioration. Prospective multicenter validation is required before these biomarkers are incorporated into clinical risk models or used to guide management decisions.

## 1. Introduction

Stanford type A acute aortic dissection (TAAD) is one of the most catastrophic cardiovascular emergencies, characterized by an intimal tear in the ascending aorta leading to life-threatening complications such as cardiac tamponade, aortic rupture, or end-organ ischemia [1,2]. Despite advances in surgical techniques and perioperative management, the Postop mortality rate in TAAD remains considerably high, ranging between 15% and 30% [3,4]. Early identification of prognostic markers associated with mortality is therefore crucial to improve clinical outcomes and guide timely interventions.

In recent years, systemic inflammation has been increasingly recognized as a key contributor to the pathophysiology and progression of aortic dissection [5]. The inflammatory response triggered by endothelial injury and aortic wall stress plays a significant role in both the onset and Postop course of the disease [6]. Several hematologic indices derived from routine complete blood counts—such as the neutrophil-to-lymphocyte ratio (NLR), platelet-to-lymphocyte ratio (PLR), and systemic immune-inflammation index (SII)—have been proposed as simple, cost-effective, and reliable markers reflecting systemic inflammatory status in cardiovascular disorders [7,8].

Alongside inflammatory markers, serum biochemical parameters such as sodium and creatinine levels are closely related to organ perfusion, renal function, and overall hemodynamic stability in patients undergoing aortic surgery [9]. Hyponatremia and elevated creatinine have been associated with increased perioperative morbidity and mortality in various cardiac conditions, yet their combined prognostic value with hemogram-based inflammatory indices in TAAD remains insufficiently investigated [10]. Understanding the interplay between these laboratory parameters could provide deeper insights into risk stratification and Postop management.

Accordingly, the primary objective of this study was to evaluate whether preoperative PLR measured at admission was associated with 30-day mortality after emergency surgical repair for TAAD. Secondary objectives were to assess other preoperative inflammatory and biochemical markers, to evaluate postoperative biomarker measurements as indicators of evolving postoperative physiology, and to explore their associations with 2-year mortality.

## 2. Materials and Methods

### 2.1. Study Design and Patient Selection

This retrospective observational study included patients who underwent emergency surgical treatment for Stanford type A acute aortic dissection between January 2008 and December 2023 at a tertiary cardiovascular surgery center. The diagnosis was confirmed by contrast-enhanced computed tomography angiography and transthoracic echocardiography. All surgical procedures were performed at a single tertiary cardiovascular surgery center during the study period. Patients with a history of chronic dissection, active infection, malignancy, or incomplete clinical or laboratory data were excluded from the study. During the study period, all consecutive patients diagnosed with Stanford type A acute aortic dissection and undergoing emergency surgical repair were screened for eligibility. Of the 538 patients initially evaluated, 32 patients were excluded due to predefined exclusion criteria or incomplete clinical or laboratory data. After applying these criteria, 506 patients were included in the final analysis. Thirty-day mortality was considered the primary outcome, and preoperative PLR measured at admission was the principal biomarker analysis. Two-year mortality and postoperative biomarker measurements were evaluated as secondary analyses. The study cohort consisted of consecutively treated patients who underwent emergency surgical repair; however, patients who did not undergo surgery were outside the study sampling frame.

### 2.2. Ethical Approval

The study was approved by the Clinical Research Ethics Committee of Akdeniz University School of Medicine (Decision No: KAEK-917, Date: 13 December 2023). The research was conducted in accordance with the principles of the Declaration of Helsinki, and patient data were reviewed retrospectively. All participants’ personal information was kept strictly confidential, and all data were used solely for scientific and academic purposes.

### 2.3. Data Collection Method

Demographic characteristics of the patients (age, sex, body mass index, smoking status, and comorbidities), laboratory findings, surgical data, and Postop outcomes were retrospectively retrieved from the hospital information system. Perioperative variables, including cardiopulmonary bypass (CPB) duration, aortic cross-clamp time, total circulatory arrest time, extubation time, as well as intensive care unit and total hospital stay, were analyzed to evaluate the clinical course and surgical outcomes.

### 2.4. Laboratory and Biochemical Parameters

Biochemical and complete blood count results of all patients were reviewed for both the Preop and Postop periods. Preoperative laboratory measurements were obtained from blood samples collected at hospital admission prior to surgical intervention, and in cases requiring urgent resuscitation, the earliest available laboratory values recorded before surgery were used for analysis. The Preop parameters included blood urea nitrogen (BUN), creatinine, sodium, potassium, aspartate aminotransferase (AST), alanine aminotransferase (ALT), C-reactive protein (CRP), hemoglobin, hematocrit, and lactate levels. The same parameters were re-evaluated on Postop day 1 and day 7. Hemogram-based inflammatory indices were calculated from the complete blood count data, including the neutrophil-to-lymphocyte ratio (NLR), platelet-to-lymphocyte ratio (PLR), systemic immune-inflammation index (SII), and monocyte-to-lymphocyte ratio (MLR). The systemic immune-inflammation index was calculated using the following formula: SII = (Platelet × Neutrophil)/Lymphocyte. The Preop, Postop day 1, and Postop day 7 values of these parameters were recorded and statistically analyzed for their association with mortality. Postoperative analyses were performed on an available-case basis; only measurements available at the corresponding postoperative sampling time point were included, and missing values were not imputed. The numbers of available and missing observations contributing to each postoperative biomarker comparison, stratified by mortality status, are reported in the corresponding table footnotes. 

### 2.5. Surgical Characteristics and Clinical Data

All patients underwent surgery through a median sternotomy. Cardiopulmonary bypass (CPB) was established via cannulation of the right atrium and either the right femoral or axillary artery. Systemic hypothermia was applied to ensure cerebral and visceral organ protection. Following aortic cross-clamping, cardiac arrest was achieved using cold blood cardioplegia.

The surgical strategy was determined according to the location of the dissection flap and the condition of the aortic valve. Procedures such as ascending aortic replacement, the Bentall procedure, ascending aortic and aortic valve replacement, or hemi/total arch replacement were performed based on the anatomical extent of the disease. In cases requiring total arch replacement, selective antegrade cerebral perfusion under deep hypothermic circulatory arrest was employed. The durations of cardiopulmonary bypass, aortic cross-clamp, and total circulatory arrest were recorded for each patient. The amount of blood and blood products transfused, intraoperative inotropic requirements, and Postop intensive care monitoring periods were also evaluated. All patients were closely monitored in the intensive care unit with continuous hemodynamic and biochemical assessments.

Postoperative complications were defined as the occurrence of major adverse clinical events during the postoperative hospital stay, including stroke, acute kidney injury requiring renal replacement therapy, reoperation for bleeding, sepsis, or significant respiratory failure requiring prolonged mechanical ventilation. These complications were recorded throughout the postoperative hospitalization period based on clinical documentation. Laboratory parameters measured on postoperative day 1 and day 7 were obtained as part of routine postoperative monitoring, and complications occurring during the postoperative course were recorded independently of the timing of laboratory measurements. In the final analytic dataset, postoperative complications were retained only as a binary composite variable; component-specific event counts, the occurrence of multiple events in the same patient, and exact complication-onset times relative to postoperative biomarker measurements were not separately available.

### 2.6. Mortality and Follow-Up

Patients were followed up during the early postoperative period (in-hospital and 30-day) and at 2-year intervals. The relationship between mortality rates and laboratory and clinical parameters was evaluated. Causes of death were classified as multiple organ failure, bleeding, cardiac causes, sepsis, or cerebrovascular events. Follow-up information was obtained from hospital electronic medical records and outpatient clinical visit data. For patients who did not return for routine follow-up visits, survival status was verified through hospital administrative databases and available national health records when possible. Follow-up data were obtained from hospital records and outpatient clinical evaluations to ensure accuracy and completeness. Two-year vital status was available for all patients in the final analytic cohort. However, exact dates of death and individual last-follow-up dates were not consistently available.

### 2.7. Statistical Analysis

All statistical analyses were performed using IBM SPSS Statistics version 27.0 (IBM Corp., Armonk, NY, USA). Continuous variables were assessed for distributional characteristics. Variables are presented as mean ± standard deviation (SD) unless otherwise specified; because NLR, PLR, SII, and MLR showed marked right-skewness, these inflammatory indices are presented as median (interquartile range [IQR]) and are compared using the Mann–Whitney U test. Associations between biomarkers and clinical outcome parameters were evaluated using Spearman’s rank correlation analysis. The diagnostic performance of laboratory parameters and hemogram-based inflammatory indices for predicting 30-day mortality was assessed using receiver operating characteristic (ROC) analysis, with area under the curve (AUC) values and 95% confidence intervals reported. Exploratory ROC-derived cutoffs were selected by maximizing the Youden J index (sensitivity + specificity − 1). Sensitivity and specificity are reported with exact binomial 95% confidence intervals; because the thresholds were derived and evaluated in the same cohort, they were considered exploratory and not clinically validated decision thresholds. Because exact postoperative death dates were not consistently available in the retrospective dataset, formal postoperative day-1 and day-7 landmark analyses could not be performed reliably. Postoperative biomarker analyses were therefore considered secondary and interpreted as associations with evolving postoperative physiological status rather than as baseline prognostic analyses. Thirty-day mortality with preoperative PLR at admission constituted the primary analysis, whereas 2-year mortality and postoperative biomarker analyses were considered secondary. Two-year mortality was analyzed as a fixed-horizon binary outcome; exact event and censoring times were unavailable, precluding reliable Kaplan–Meier and Cox proportional-hazards analyses. Given the limited number of 30-day mortality events, a parsimonious clinical reference model was defined using age, preoperative creatinine, aortic cross-clamp time, and total circulatory arrest time. Each preoperative inflammatory index (NLR, PLR, SII, and MLR) was subsequently added separately to this reference model because of collinearity among the indices. Incremental contribution was assessed using likelihood-ratio testing and change in model discrimination (AUC). Changes in AUC between nested models were formally compared using paired DeLong tests, with 95% confidence intervals reported for AUC differences. Multicollinearity was assessed using variance inflation factors (VIFs), with VIF values >5 considered indicative of potentially important collinearity. Internal validity of the primary PLR-augmented model was evaluated using 1000 nonparametric bootstrap resamples, with optimism estimated from the difference between bootstrap-sample and original-sample performance. Optimism-corrected AUC, Brier score, calibration intercept, and calibration slope were calculated, and model calibration was assessed graphically. Available clinical variables also included sex, BMI, smoking status, comorbidity status, ejection fraction, aortic regurgitation grade, ascending aortic diameter, dissection type, surgical procedure, CPB duration, transfusion variables, and inotropic requirement; preoperative shock, tamponade, malperfusion, neurological status, aortic rupture, and prior resuscitation were not captured as separate structured variables in the analytic dataset. Preoperative PLR in relation to 30-day mortality constituted the primary biomarker analysis. No multiplicity adjustment was applied to this single primary analysis; all remaining biomarker, time-point, and outcome comparisons were considered exploratory, and their unadjusted *p* values should be interpreted as hypothesis-generating rather than confirmatory. Adjusted odds ratios (ORs) with 95% confidence intervals (CIs) were reported. All statistical tests were two-tailed, and *p*-values < 0.05 were considered statistically significant.

## 3. Results

When preoperative and postoperative laboratory and clinical variables were compared according to 30-day mortality status, no significant differences were observed between the groups in terms of age, BMI, cardiopulmonary bypass duration, extubation time, or ascending aortic diameter (*p* > 0.05). In contrast, both aortic cross-clamp time and total circulatory arrest time were significantly longer in patients who died within 30 days (*p* < 0.001 for both). While preoperative BUN and sodium levels did not differ significantly between the groups, preoperative creatinine was markedly higher in the mortality group (*p* = 0.008). In the postoperative period, creatinine levels remained significantly elevated among non-survivors (*p* = 0.003), whereas sodium and potassium levels showed no significant differences. Postoperative lactate levels were higher in patients who died and reached statistical significance (*p* = 0.029). In addition to the primary preoperative PLR analysis, exploratory comparisons showed higher NLR, SII, and MLR values, as well as higher postoperative inflammatory indices, in patients who died within 30 days. All inflammatory markers were significantly elevated in the mortality group (all *p* < 0.001), with the exception of preoperative MLR (*p* = 0.011), postoperative day 1 MLR (*p* = 0.007), and postoperative day 7 MLR (*p* = 0.010) (Table 1).

Spearman correlation analysis was performed to evaluate the relationships between preoperative and postoperative biomarkers and clinical outcome parameters. The majority of the examined variables did not demonstrate significant correlations with intensive care unit stay, total hospital stay, or extubation time. Although preoperative creatinine showed a weak positive correlation with ICU stay (r = 0.086), this association did not reach statistical significance (*p* = 0.053). Likewise, preoperative sodium and lactate levels did not exhibit significant correlations with any of the three clinical outcomes (all *p* > 0.05). Similarly, postoperative creatinine, sodium, and lactate levels were not significantly associated with ICU stay, hospital stay, or extubation duration (all *p* > 0.05). Regarding hemogram-derived inflammatory indices, most preoperative and postoperative NLR, PLR, SII, and MLR values showed no significant correlations with the evaluated outcome parameters. However, NLR demonstrated a weak but statistically significant positive correlation with hospital length of stay (preoperative NLR: r = 0.108, *p* = 0.015; postoperative day 1 NLR: r = 0.106, *p* = 0.017; postoperative day 7 NLR: r = 0.104, *p* = 0.020). Although the magnitudes of these correlations were low, they met the threshold for statistical significance (Table 2).

When preoperative and postoperative laboratory parameters were compared according to the presence of postoperative complications, none of the evaluated biochemical markers demonstrated a statistically significant difference between the groups. Preoperative creatinine, sodium, and lactate levels were comparable in patients with and without complications (all *p* > 0.05). Similarly, postoperative creatinine, sodium, and lactate values did not differ significantly between the groups (all *p* > 0.05). Assessment of hemogram-derived inflammatory indices showed that preoperative and postoperative NLR, PLR, SII, and MLR levels were also similar between patients with and without complications (all *p* > 0.05). No discernible separation between the groups was observed for any of these indices at the preoperative, postoperative day 1, or postoperative day 7 time points (Table 3).

**Figure 1:**
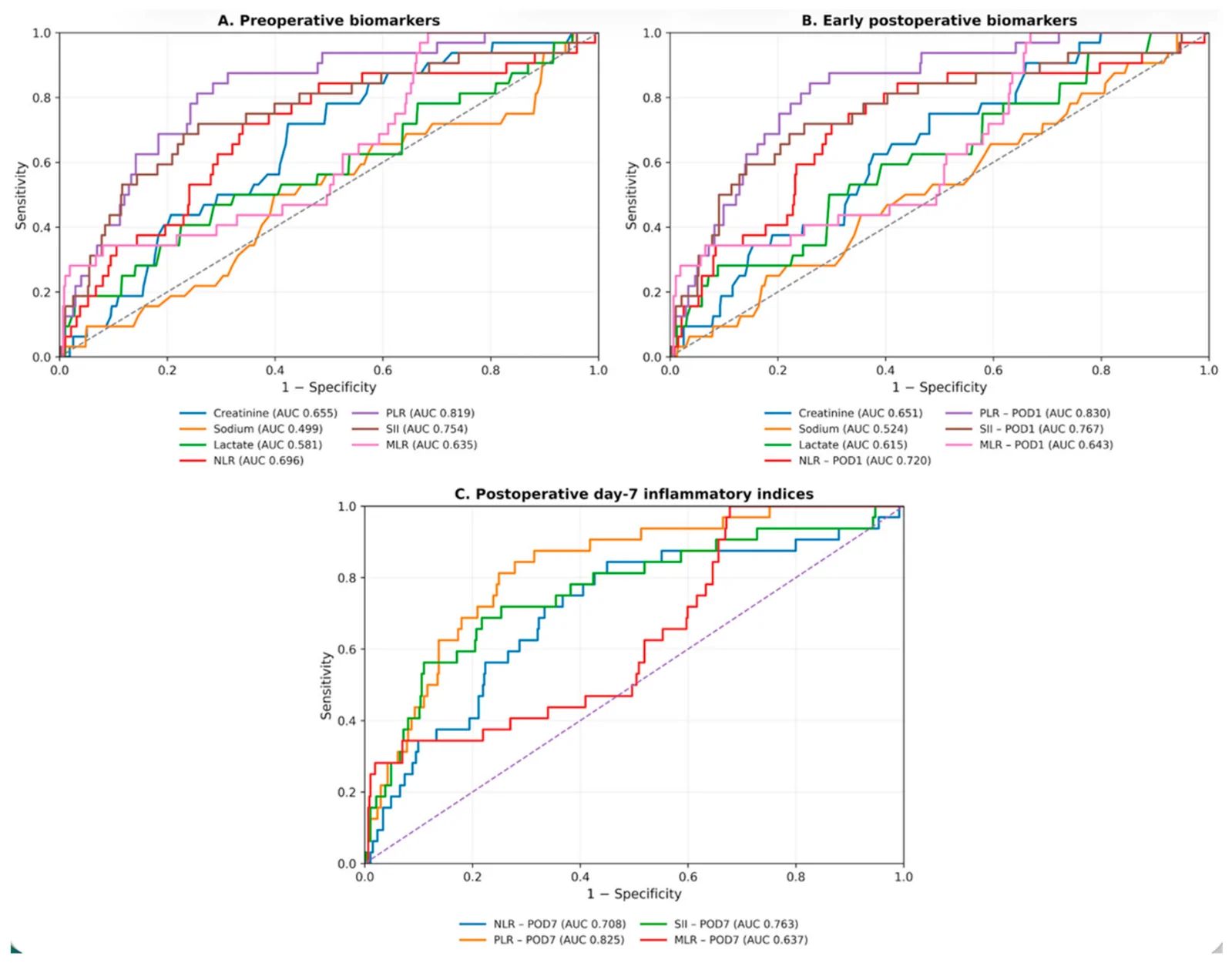
Receiver operating characteristic (ROC) curves of inflammatory indices and biochemical markers for 30-day mortality. (**A**) Preoperative biomarkers: creatinine, sodium, lactate, NLR, PLR, SII, and MLR. (**B**) Early postoperative biomarkers: creatinine, sodium, lactate, and postoperative day-1 NLR, PLR, SII, and MLR. (**C**) Postoperative day-7 inflammatory indices: NLR, PLR, SII, and MLR. The dashed diagonal line represents no discrimination. AUC values are shown in the panel legends.

For preoperative creatinine, the AUC was 0.655, indicating a moderate discriminatory ability for predicting 30-day mortality (*p* = 0.003). Postoperative creatinine demonstrated a similar predictive performance, with an AUC of 0.651 (*p* = 0.004). In contrast, preoperative and postoperative sodium levels yielded AUC values of 0.499 and 0.524, respectively, demonstrating no diagnostic contribution (both *p* > 0.05). Preoperative lactate was not a significant predictor of mortality (AUC = 0.581, *p* = 0.126), whereas postoperative lactate exhibited modest but statistically significant discriminatory capacity (AUC = 0.615, *p* = 0.029). Among the hemogram-derived inflammatory indices, preoperative NLR showed an AUC of 0.696, while postoperative day 1 and day 7 NLR values exhibited improved discriminatory performance (AUC = 0.720 and 0.708, respectively; all *p* < 0.001). PLR demonstrated AUC values of 0.819 preoperatively, 0.830 on postoperative day 1, and 0.825 on postoperative day 7 (all *p* < 0.001). SII also demonstrated robust discriminatory ability, with AUC values of 0.754 (preoperative), 0.767 (postoperative day 1), and 0.763 (postoperative day 7), all of which were statistically significant (*p* < 0.001). Although MLR exhibited lower discriminatory performance, it nonetheless provided statistically significant discrimination at all measured time points (AUC range: 0.635–0.643; all *p* < 0.001) (Table 4, Figure 1).

When preoperative and postoperative variables were compared according to 2-year mortality status, both aortic cross-clamp duration and total circulatory arrest time were found to be significantly longer in patients who died during follow-up (*p* < 0.001 for both). In contrast, cardiopulmonary bypass duration did not differ significantly between the groups (*p* = 0.238). Intensive care unit stay, total hospital stay, and extubation time likewise showed no significant variation according to mortality status (all *p* > 0.05). With respect to renal function indicators, preoperative creatinine levels were significantly higher in the mortality group (*p* = 0.011), and this difference persisted in the postoperative period, with postoperative creatinine also elevated among non-survivors (*p* = 0.002). Preoperative sodium levels did not differ significantly between groups (*p* = 0.266). In contrast, for 2-year mortality, both preoperative and postoperative lactate levels were substantially higher in patients who died, demonstrating strong statistical significance (both *p* < 0.001). Analysis of hemogram-derived inflammatory indices demonstrated that NLR, PLR, SII, and MLR values were significantly elevated in the mortality group at all preoperative and postoperative time points (all *p* < 0.001). In exploratory secondary analyses, NLR, PLR, SII, and MLR values were higher in the 2-year mortality group at all measured time points (all *p* < 0.001) (Table 5).

The clinical reference model comprising age, preoperative creatinine, aortic cross-clamp time, and total circulatory arrest time demonstrated an AUC of 0.840 for 30-day mortality. Addition of preoperative PLR increased the AUC to 0.883 (ΔAUC = 0.043, 95% CI 0.010–0.075; DeLong *p* = 0.010), while addition of SII increased the AUC to 0.872 (ΔAUC = 0.032, 95% CI 0.006–0.059; DeLong *p* = 0.018). Both PLR and SII also significantly improved model fit by likelihood-ratio testing (*p* < 0.001 and *p* = 0.002, respectively). The difference in AUC between the PLR- and SII-augmented models was not statistically significant (ΔAUC = 0.011, 95% CI −0.007 to 0.028; *p* = 0.242). Preoperative PLR remained independently associated with 30-day mortality (adjusted OR 1.004, 95% CI 1.002–1.007; *p* < 0.001) (Table 6).

**Figure 2:**
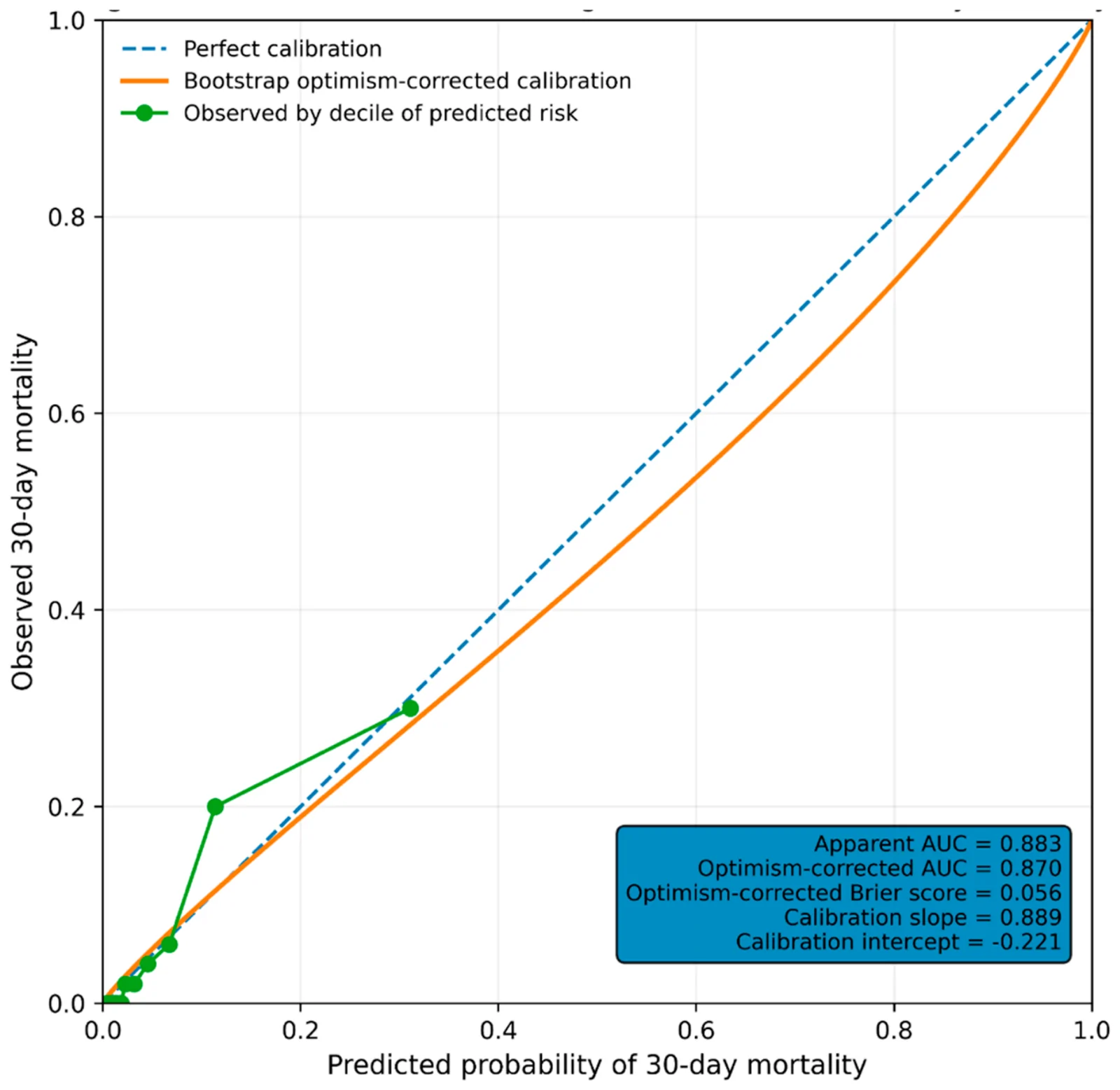
Calibration of the primary PLR-augmented multivariable model for 30-day mortality. Observed 30-day mortality rates are plotted against predicted probabilities across groups of predicted risk, with the diagonal line representing perfect calibration. Bootstrap internal validation with 1000 resamples yielded an optimism-corrected AUC of 0.870, Brier score of 0.056, calibration slope of 0.889, and calibration intercept of −0.221.

Collinearity diagnostics showed no meaningful multicollinearity in the primary PLR-augmented model (VIFs: age 1.03, preoperative creatinine 1.03, cross-clamp time 1.08, total circulatory arrest time 1.08, and PLR 1.06); the correlation between cross-clamp and circulatory-arrest times was modest (r = 0.20). In 1000-bootstrap internal validation, the apparent AUC of 0.883 was optimism-corrected to 0.870; the optimism-corrected Brier score was 0.056, calibration slope was 0.889, and calibration intercept was −0.221 (Figure 2), indicating modest model optimism.

## 4. Discussion

The demonstration in our study that both preoperative and postoperative hemogram-derived inflammatory indices (NLR, PLR, SII, and MLR) are strongly associated with mortality after TAAD surgery is consistent with accumulating evidence highlighting the central role of inflammation in the pathophysiology of aortic dissection. In a large cohort of 691 patients, Xie et al. reported that preoperative SIRI, NLR, MLR, and SII were independently associated with both short-term major adverse events and long-term aorta-related adverse events [11]. Similarly, Xin et al., in a cohort of 597 patients, observed that elevated admission NLR, MLR, and SII levels were robust predictors of in-hospital mortality [12]. In line with these findings, our results showed significant differences in all inflammatory indices between survivors and non-survivors, with PLR and SII demonstrating significant discrimination in the univariable ROC analyses. These observations further support the concept that inflammation-driven immune activation is a key determinant of prognosis in acute aortic dissection. Moreover, our findings correspond with those of Vrsalović, whose meta-analysis confirmed the prognostic relevance of inflammatory biomarkers in aortic dissection [13].

Preoperative PLR was the primary biomarker analysis for 30-day mortality, whereas the remaining inflammatory-index, postoperative, and 2-year analyses were exploratory. Within these exploratory analyses, NLR, SII, and MLR also differed between survivors and non-survivors. These findings align with prior evidence suggesting that hematologic inflammatory ratios capture the underlying inflammation–immune imbalance characteristic of dissection pathophysiology. For example, Ustaalıoğlu et al. reported that elevated NLR was significantly associated with mortality in TAAD patients [14], while Bedel and Selvi similarly identified both NLR and PLR as strong predictors of in-hospital mortality in type A dissection [15]. Consistent with these results, Xie’s 2024 cohort study demonstrated that NLR, MLR, and SII were independent predictors of both major adverse events and long-term aorta-related adverse events [11]. These observations correspond well with our ROC analysis in which PLR and SII showed particularly high discriminatory performance (AUC 0.819–0.830). Conversely, some studies by Sbaourini, Liu, and Suzuki have reported weaker predictive performance of individual inflammatory ratios; however, these discrepancies likely reflect smaller sample sizes, heterogeneity in surgical techniques, and differences in perioperative management strategies [16,17,18].

The multivariable findings should be interpreted in light of incomplete capture of several established markers of TAAD severity. Preoperative shock, cardiac tamponade, malperfusion, neurological status, aortic rupture, and prior resuscitation are recognized clinical determinants of adverse outcome after TAAD repair [2,19]. However, these variables were not retained as separate structured variables in the analytic dataset and therefore could not be included in the adjusted models. In addition, with only 32 deaths within 30 days, substantially expanding the number of covariates would have increased the risk of model overfitting and instability; we therefore used a parsimonious clinical reference model based on variables that were reliably available across the cohort. Residual confounding from unmeasured disease severity remains possible, and the adjusted biomarker associations should not be interpreted as fully independent of all established TAAD risk factors.

In our study, both preoperative and postoperative creatinine levels were significantly associated with early and long-term mortality, underscoring the role of renal dysfunction as a perioperative risk marker in TAAD. This finding aligns with the work of Wu et al., who, in a cohort of 609 patients, demonstrated that elevated preoperative serum creatinine independently increased 30-day mortality, while the use of continuous renal replacement therapy partially mitigated the adverse effects of severe renal impairment [20]. Similarly, Yang’s 2020 analysis identified postoperative day-1 creatinine as a powerful predictor of one-year mortality following TAAD repair [21]. These observations correspond closely with our results, in which creatinine levels were significantly higher in both the 30-day and 2-year mortality groups. Furthermore, the studies by Cai and colleagues indicate that renal dysfunction often reflects a combination of impaired organ perfusion and heightened inflammatory burden, thereby serving as an integrated marker of dissection severity [22,23].

The association between postoperative lactate levels and mortality should be interpreted as reflecting evolving postoperative physiological stress and impaired tissue perfusion rather than baseline prognostic risk. This finding is consistent with prior evidence indicating that elevated lactate is a surrogate for impaired organ perfusion and heightened metabolic stress. Hanna et al. reported that tissue hypoxia and systemic inflammatory activation increase in parallel with dissection severity, contributing directly to adverse outcomes [24]. Similarly, Lin et al. reported that postoperative lactate was independently associated with in-hospital mortality after acute type A aortic dissection surgery [25]. While preoperative lactate showed limited discriminative ability in our cohort, elevated postoperative lactate may reflect the effects of intraoperative ischemia–reperfusion injury and subsequent hemodynamic instability. Collectively, these findings highlight postoperative lactate as a sensitive indicator of global perfusion adequacy and a valuable early marker of postoperative deterioration.

In our study, the lack of a significant association between inflammatory indices and postoperative complications suggests that systemic inflammation may be more closely linked to the profound physiological deterioration leading to mortality rather than to the development of early, discrete postoperative morbidities. This interpretation is consistent with the findings of Xie et al. [11], who demonstrated that composite inflammatory scores—such as SIRI and SII—were strong predictors of major adverse events, including severe postoperative complications such as stroke, myocardial infarction, acute kidney injury, and reoperation, as well as long-term aorta-related adverse outcomes. However, they also noted that postoperative complications themselves were influenced by multifactorial and heterogeneous mechanisms rather than inflammation alone [11]. Similarly, studies by Eghbalzadeh and Sbaourini have shown that although inflammatory markers reliably predict mortality in TAAD, their relationship with postoperative complication profiles is inconsistent or weak, likely due to the complex interplay of surgical, anatomical, and perioperative management factors [17,26]. Taken together, these observations support the notion that heightened inflammation predominantly reflects severe systemic pathological processes—such as renal impairment, metabolic dysregulation, and systemic inflammatory response syndrome—that contribute directly to fatal outcomes rather than to the occurrence of early postoperative complications.

Taken together, preoperative inflammatory indices were associated with mortality, whereas postoperative inflammatory indices, creatinine, and lactate may reflect evolving postoperative physiological risk. This interpretation is consistent with previous studies by Wu, Xin, and Xie reporting prognostic associations between inflammatory or organ-function markers and adverse outcomes in TAAD [11,12,20], as well as with reports by Cai and Suzuki linking inflammatory and perfusion-related abnormalities with postoperative outcomes [18,22]. However, the present retrospective data demonstrate association and discrimination only and do not establish that biomarker-guided management improves clinical decision-making or patient outcomes. Prospective multicenter studies are required to determine whether incorporation of these biomarkers into clinical risk models provides clinically useful incremental value.

A potential hypothesis for future prospective interventional studies is whether patients with a high inflammatory burden may benefit from adjunct anti-inflammatory strategies, including hemoadsorption; however, this was not evaluated in the present study [27,28].

The observed 30-day mortality rate in our cohort was 6.3% (32/506), which is lower than that reported in many surgical TAAD series. This finding should be interpreted in the context of the study design and patient-selection process. By design, the cohort included only patients who reached emergency surgery and had sufficient clinical and laboratory data for analysis; patients who did not undergo surgery were outside the sampling frame, and 32 screened patients were excluded because of predefined exclusion criteria or incomplete data. In addition, single-center referral patterns, case selection, and institutional experience may have influenced the observed mortality rate. Therefore, selection and referral bias cannot be excluded, and the mortality rate observed in this cohort should not be generalized to all patients presenting with acute TAAD.

This study has several limitations. First, its retrospective and single-center design may introduce potential selection bias and limit the generalizability of the findings. Because the data were obtained from a single tertiary cardiovascular surgery center, institutional surgical strategies, perioperative management protocols, and referral patterns may have influenced the observed outcomes. In addition, patient-level calendar year of surgery was not retained as a separate analyzable variable in the final analytic dataset; therefore, annual case and mortality distributions, adjustment for surgical era, and era-stratified sensitivity analyses could not be performed, and temporal changes in surgical and perioperative practice remain a potential source of residual confounding. Second, inflammatory indices and biochemical markers were measured at predefined perioperative time points, which prevented assessment of their dynamic temporal changes throughout the perioperative period. In addition, exact dates of death and individual last-follow-up dates were not consistently available, precluding reliable landmark and long-term time-to-event analyses; therefore, postoperative biomarker associations and fixed-horizon 2-year mortality findings should be interpreted cautiously. In addition, several established TAAD severity variables, including preoperative shock, tamponade, malperfusion, neurological status, aortic rupture, and prior resuscitation, were not captured as separate structured variables and could not be included in multivariable adjustment. Third, although multivariable analyses were performed to adjust for major clinical variables, residual confounding from unmeasured perioperative factors—such as detailed hemodynamic management, transfusion strategies, or postoperative care protocols—cannot be completely excluded. Fourth, postoperative complications were analyzed as a binary outcome without stratification by severity or organ-specific classification, which may have limited the detection of associations between inflammatory indices and specific postoperative morbidities. Finally, hemogram-based inflammatory indices are surrogate inflammatory markers that may be influenced by multiple clinical conditions and should therefore be interpreted cautiously in conjunction with other perioperative risk factors. In addition, because the number of mortality events was relatively limited, the regression estimates should be interpreted cautiously, as residual confounding and potential model instability cannot be entirely excluded. Because postoperative complications were available only as a binary composite variable, component-specific analyses, assessment of multiple events per patient, and evaluation of the temporal relationship between biomarker measurement and complication onset could not be performed.

## 5. Conclusions

In this retrospective cohort, preoperative PLR was independently associated with 30-day mortality, while other inflammatory indices were also associated with mortality in exploratory analyses. Postoperative inflammatory indices, creatinine, and lactate should be interpreted as markers of evolving postoperative physiological deterioration rather than as baseline predictors. Prospective multicenter validation is required before these biomarkers can be incorporated into clinical risk models or used to guide management decisions.

## Figures and Tables

**Table 1:** Comparison of Preop and Postop laboratory and clinical variables according to 30-day mortality.

Variable		No (*n* = 474)	Yes (*n* = 32)	*p*-Value
Age (years)		59.4 ± 10.8	60.1 ± 9.9	0.736 ^b^
BMI (kg/m^2^)		27.3 ± 4.0	26.0 ± 4.1	0.068 ^a^
CP Bypass Time (min)		174.3 ± 35.4	171.7 ± 29.3	0.681 ^a^
Aortic Cross-Clamp Time (min)		103.7 ± 28.9	128.4 ± 24.6	**<0.001 ^a^**
Total Circulatory Arrest Time (min)		18.0 ± 1.7	19.5 ± 1.4	**<0.001 ^a^**
Extubation Time (hours)		14.1 ± 5.7	12.9 ± 4.0	0.171 ^b^
Ascending Aortic Diameter (mm)		47.3 ± 6.1	47.5 ± 6.7	0.885 ^a^
Type of Surgery	Ascending	250 (52.7)	10 (31.3)	**0.015**
	Ascending + AVR	136 (28.7)	9 (28.1)	
	Bentall	54 (11.4)	6 (18.8)	
	Hemi Aortic Arch	22 (4.6)	4 (12.5)	
	Total Aortic Arch	12 (2.5)	3 (9.4)	
Preop BUN (mg/dL)		26.9 ± 9.6	25.9 ± 9.1	0.505 ^b^
Preop Creatinine (mg/dL)		1.3 ± 0.5	1.6 ± 0.4	**0.008 ^a^**
Preop Sodium (mmol/L)		140.6 ± 3.2	140.6 ± 3.3	0.984 ^b^
Preop Potassium (mmol/L)		4.2 ± 0.5	4.1 ± 0.5	0.422 ^b^
Postop BUN (mg/dL)		30.3 ± 12.5	28.1 ± 11.0	0.351 ^a^
Postop Creatinine (mg/dL)		1.6 ± 0.6	1.9 ± 0.5	**0.003 ^a^**
Postop Sodium (mmol/L)		140.9 ± 3.9	141.2 ± 3.7	0.609 ^a^
Postop Potassium (mmol/L)		4.2 ± 0.7	4.1 ± 0.6	0.643 ^b^
Preop Lactate (mmol/L)		2.4 ± 0.9	2.8 ± 1.1	0.126 ^b^
Postop Lactate (mmol/L)		3.0 ± 1.2	3.5 ± 1.3	**0.029 ^b^**
NLR—Preop		4.9 (3.7–7.0)	7.1 (5.6–10.6)	**<0.001 ^b^**
NLR—Postop Day 1		5.4 (4.1–7.9)	8.2 (6.8–12.7)	**<0.001 ^b^**
NLR—Postop Day 7		3.9 (2.9–5.6)	5.9 (4.6–8.7)	**<0.001 ^b^**
PLR—Preop		125.8 (92.9–179.0)	257.0 (182.5–351.2)	**<0.001 ^b^**
PLR—Postop Day 1		140.8 (104.2–203.4)	295.2 (217.5–406.5)	**<0.001 ^b^**
PLR—Postop Day 7		101.0 (74.4–143.6)	210.1 (146.8–298.2)	**<0.001 ^b^**
SII—Preop		1034.3 (684.0–1670.5)	2282.9 (1274.3–3092.5)	**<0.001 ^b^**
SII—Postop Day 1		1170.1 (762.6–1886.6)	2754.3 (1486.7–3636.1)	**<0.001 ^b^**
SII—Postop Day 7		823.7 (548.3–1321.4)	1914.0 (1015.2–2627.3)	**<0.001 ^b^**
MLR—Preop		0.65 (0.11–2.26)	0.68 (0.31–9.80)	**0.011 ^b^**
MLR—Postop Day 1		0.74 (0.13–2.53)	0.80 (0.38–11.66)	**0.007 ^b^**
MLR—Postop Day 7		0.53 (0.09–1.81)	0.55 (0.27–7.74)	**0.010 ^b^**

Data are presented as mean ± standard deviation unless otherwise specified; NLR, PLR, SII, and MLR are presented as median (interquartile range [IQR]). ^a^: Independent-samples *t* test. ^b^: Mann–Whitney U test. Statistically significant *p* values are shown in bold. No observations were missing for the postoperative biomarkers included in the analysis. For all postoperative creatinine, sodium, lactate, NLR, PLR, SII, and MLR measurements, including POD1 and POD7 where applicable, available observations were *n* = 474/32 and missing observations were *n* = 0/0 for 30-day survivors/non-survivors, respectively. Abbreviations: BUN, blood urea nitrogen; NLR, neutrophil-to-lymphocyte ratio; PLR, platelet-to-lymphocyte ratio; SII, systemic immune-inflammation index; MLR, monocyte-to-lymphocyte ratio; Preop, preoperative; Postop, postoperative; CP, cardiopulmonary.

**Table 2:** Correlation between preoperative and postoperative biomarkers and clinical outcome parameters.

Variable	ICU Stay (Days)	*p*	Hospital Stay (Days)	*p*	Extubation Time (Hours)	*p*
Preop Creatinine (mg/dL)	0.086	0.053	0.058	0.190	–0.039	0.380
Preop Sodium (mmol/L)	0.005	0.916	–0.065	0.143	0.037	0.408
Preop Lactate (mmol/L)	0.009	0.838	0.034	0.447	–0.070	0.117
Postop Creatinine (mg/dL)	0.050	0.263	–0.032	0.474	0.009	0.840
Postop Sodium (mmol/L)	–0.016	0.716	–0.057	0.198	0.030	0.495
Postop Lactate (mmol/L)	–0.043	0.330	0.018	0.686	0.039	0.383
NLR—Preop	–0.014	0.745	0.108	**0.015**	–0.054	0.227
NLR—Postop Day 1	–0.016	0.712	0.106	**0.017**	–0.061	0.172
NLR—Postop Day 7	–0.013	0.764	0.104	**0.020**	–0.053	0.235
PLR—Preop	–0.003	0.944	0.057	0.199	–0.070	0.114
PLR—Postop Day 1	–0.002	0.963	0.056	0.208	–0.066	0.137
PLR—Postop Day 7	–0.004	0.920	0.063	0.159	–0.068	0.126
SII—Preop	–0.002	0.971	0.058	0.192	–0.068	0.128
SII—Postop Day 1	–0.001	0.979	0.056	0.205	–0.066	0.140
SII—Postop Day 7	–0.004	0.926	0.059	0.185	–0.070	0.115
MLR—Preop	0.052	0.239	0.004	0.937	–0.056	0.207
MLR—Postop Day 1	0.052	0.243	0.004	0.924	–0.055	0.216
MLR—Postop Day 7	0.054	0.227	0.001	0.976	–0.055	0.220

Data are presented as Spearman’s correlation coefficient (r) and corresponding *p* values. Statistically significant *p* values are shown in bold. Abbreviations: NLR, neutrophil-to-lymphocyte ratio; PLR, platelet-to-lymphocyte ratio; SII, systemic immune-inflammation index; MLR, monocyte-to-lymphocyte ratio; Preop, preoperative; Postop, postoperative; ICU, intensive care unit.

**Table 3:** Comparison of preoperative and postoperative laboratory indices according to postoperative complications.

Variable	No (*n* = 336)	Yes (*n* = 170)	*p*-Value
Preop Creatinine (mg/dL)	1.4 ± 0.5	1.3 ± 0.5	0.267 ^a^
Preop Sodium (mmol/L)	140.7 ± 3.0	140.3 ± 3.4	0.094 ^b^
Postop Creatinine (mg/dL)	1.6 ± 0.6	1.5 ± 0.5	0.090 ^a^
Postop Sodium (mmol/L)	141.0 ± 3.5	140.7 ± 4.4	0.384 ^a^
Preop Lactate (mmol/L)	2.4 ± 0.9	2.5 ± 0.9	0.910 ^b^
Postop Lactate (mmol/L)	3.1 ± 1.2	3.0 ± 1.3	0.416 ^b^
NLR—Preop	5.2 (3.7–7.1)	4.9 (3.6–7.2)	0.773 ^b^
NLR—Postop Day 1	5.7 (4.3–8.1)	5.4 (4.0–8.3)	0.754 ^b^
NLR—Postop Day 7	4.1 (3.0–5.7)	3.8 (2.8–5.9)	0.710 ^b^
PLR—Preop	126.2 (92.6–183.7)	133.7 (98.4–194.8)	0.255 ^b^
PLR—Postop Day 1	142.4 (103.6–210.4)	151.0 (110.0–221.1)	0.263 ^b^
PLR—Postop Day 7	101.2 (74.1–148.6)	107.5 (80.1–159.2)	0.222 ^b^
SII—Preop	1066.9 (695.3–1737.8)	1039.9 (700.1–1879.1)	0.774 ^b^
SII—Postop Day 1	1199.3 (776.2–1966.4)	1170.3 (786.8–2079.6)	0.712 ^b^
SII—Postop Day 7	852.4 (552.3–1399.3)	823.7 (564.9–1516.8)	0.748 ^b^
MLR—Preop	0.62 (0.12–2.21)	0.72 (0.14–2.81)	0.313 ^b^
MLR—Postop Day 1	0.71 (0.13–2.49)	0.81 (0.16–3.18)	0.323 ^b^
MLR—Postop Day 7	0.51 (0.09–1.76)	0.57 (0.11–2.27)	0.340 ^b^

Data are presented as mean ± standard deviation unless otherwise specified; NLR, PLR, SII, and MLR are presented as median (interquartile range [IQR]). ^a^: Independent-samples *t* test. ^b^: Mann–Whitney U test. Statistically significant *p* values are shown in bold. Abbreviations: NLR, neutrophil-to-lymphocyte ratio; PLR, platelet-to-lymphocyte ratio; SII, systemic immune-inflammation index; MLR, monocyte-to-lymphocyte ratio; Preop, preoperative; Postop, postoperative; Postoperative complications were analyzed as a binary composite outcome because component-specific event data and event timing were not available in the final analytic dataset.

**Table 4:** Diagnostic performance of hemogram-based inflammatory indices and sodium, creatinine and lactate levels for 30-day mortality.

Test Result Variable	AUC (95% CI)	Exploratory Cut-Off	Sensitivity, % (95% CI)	Specificity, % (95% CI)	*p*-Value
Preop Creatinine (mg/dL)	**0.655 (0.569–0.741)**	**1.40**	**72 (53–86)**	**58 (53–62)**	**0.003**
Preop Sodium (mmol/L)	**0.499 (0.394–0.604)**	**141.6**	**50 (32–68)**	**60 (56–65)**	**0.984**
Preop Lactate (mmol/L)	**0.581 (0.469–0.692)**	**2.93**	**47 (29–65)**	**71 (67–75)**	**0.126**
Postop Cr (mg/dL)	**0.651 (0.564–0.737)**	**1.56**	**75 (57–89)**	**52 (47–56)**	**0.004**
Postop Sodium (mmol/L)	**0.524 (0.422–0.626)**	**142.2**	**44 (26–62)**	**65 (60–69)**	**0.651**
Postop Lactate (mmol/L)	**0.615 (0.514–0.716)**	**3.70**	**50 (32–68)**	**70 (66–75)**	**0.029**
NLR—Preop	**0.696 (0.598–0.794)**	**6.08**	**72 (53–86)**	**66 (62–70)**	**<0.001**
NLR—Postop Day 1	**0.720 (0.623–0.816)**	**6.13**	**84 (67–95)**	**58 (53–62)**	**<0.001**
NLR—Postop Day 7	**0.708 (0.611–0.805)**	**4.21**	**84 (67–95)**	**55 (50–60)**	**<0.001**
PLR—Preop	**0.819 (0.750–0.889)**	**156.2**	**88 (71–96)**	**69 (64–73)**	**<0.001**
PLR—Postop Day 1	**0.830 (0.766–0.895)**	**200.3**	**84 (67–95)**	**74 (70–78)**	**<0.001**
PLR—Postop Day 7	**0.825 (0.758–0.892)**	**136.6**	**84 (67–95)**	**72 (68–76)**	**<0.001**
SII—Preop	**0.754 (0.658–0.851)**	**1603.7**	**72 (53–86)**	**74 (70–78)**	**<0.001**
SII—Postop Day 1	**0.767 (0.672–0.861)**	**1888.6**	**72 (53–86)**	**75 (71–79)**	**<0.001**
SII—Postop Day 7	**0.763 (0.667–0.858)**	**1468.2**	**69 (50–84)**	**78 (74–82)**	**<0.001**
MLR—Preop	**0.635 (0.537–0.733)**	**0.220**	**100 (89–100)**	**32 (27–36)**	**0.011**
MLR—Postop Day 1	**0.643 (0.547–0.739)**	**0.295**	**100 (89–100)**	**33 (29–38)**	**0.007**
MLR—Postop Day 7	**0.637 (0.539–0.734)**	**0.199**	**100 (89–100)**	**32 (28–37)**	**0.010**

Data are presented as area under the curve (AUC) with 95% confidence intervals, exploratory ROC-derived cutoffs, sensitivity, and specificity. Cutoffs were selected by maximizing the Youden J index within the study cohort. Sensitivity and specificity are presented with exact binomial 95% confidence intervals. *p* values refer to testing the AUC against the null value of 0.50. Because the thresholds were derived and evaluated in the same cohort, they should be considered exploratory and require independent validation before clinical application.

**Table 5:** Comparison of preoperative and postoperative variables according to 2-year mortality status.

Variable	No (*n* = 416)	Yes (*n* = 90)	*p*
CP Bypass Time (min)	175.0 ± 35.4	170.2 ± 33.3	0.238 ^a^
Aortic Cross-Clamp (min)	101.2 ± 27.3	124.4 ± 30.7	**<0.001 ^a^**
Total Circulatory Arrest (min)	17.8 ± 1.5	19.6 ± 1.6	**<0.001 ^a^**
ICU Stay (days)	4.8 ± 2.1	4.8 ± 2.3	0.958 ^b^
Hospital Stay (days)	11.6 ± 6.5	11.1 ± 5.1	0.789 ^b^
Extubation Time (hours)	14.2 ± 5.7	13.0 ± 5.3	0.069 ^b^
Preop Creatinine (mg/dL)	1.3 ± 0.5	1.5 ± 0.5	**0.011 ^a^**
Preop Sodium (mmol/L)	140.6 ± 3.1	140.3 ± 3.3	0.266 ^b^
Preop Lactate (mmol/L)	2.4 ± 0.9	2.8 ± 1.0	**<0.001 ^b^**
Postoperative Cr (mg/dL)	1.5 ± 0.5	1.7 ± 0.6	**0.002 ^a^**
Postoperative Sodium (mmol/L)	141.0 ± 3.8	140.5 ± 3.9	0.303 ^a^
Postoperative Lactate (mmol/L)	2.9 ± 1.2	3.5 ± 1.3	**<0.001 ^b^**
NLR—Preop	4.5 (3.6–6.4)	9.0 (6.5–11.0)	**<0.001 ^b^**
NLR—Postop Day 1	5.0 (4.0–7.1)	10.5 (7.6–13.0)	**<0.001 ^b^**
NLR—Postop Day 7	3.6 (2.8–5.1)	7.2 (5.1–9.0)	**<0.001 ^b^**
PLR—Preop	118.0 (90.5–154.7)	262.1 (188.9–337.8)	**<0.001 ^b^**
PLR—Postop Day 1	133.4 (100.0–174.3)	310.1 (225.6–396.3)	**<0.001 ^b^**
PLR—Postop Day 7	96.2 (72.5–128.9)	216.2 (154.5–274.9)	**<0.001 ^b^**
SII—Preop	976.1 (645.0–1398.8)	2282.9 (1566.2–3170.6)	**<0.001 ^b^**
SII—Postop Day 1	1092.5 (714.0–1533.9)	2742.4 (1824.8–3684.1)	**<0.001 ^b^**
SII—Postop Day 7	770.2 (520.1–1105.1)	1914.0 (1245.7–2598.1)	**<0.001 ^b^**
MLR—Preop	0.67 (0.10–2.21)	0.64 (0.34–6.05)	**<0.001 ^b^**
MLR—Postop Day 1	0.75 (0.11–2.48)	0.73 (0.40–7.14)	**<0.001 ^b^**
MLR—Postop Day 7	0.53 (0.08–1.77)	0.52 (0.28–4.97)	**<0.001 ^b^**

Data are presented as mean ± standard deviation unless otherwise specified; NLR, PLR, SII, and MLR are presented as median (interquartile range [IQR]). ^a^: Independent-samples *t* test. ^b^: Mann–Whitney U test. Statistically significant *p* values are shown in bold. No observations were missing for the postoperative biomarkers included in the analysis. For all postoperative creatinine, sodium, lactate, NLR, PLR, SII, and MLR measurements, including POD1 and POD7 where applicable, available observations were *n* = 416/90 and missing observations were *n* = 0/0 for 2-year survivors/non-survivors, respectively. Abbreviations: CP, cardiopulmonary; Preop, preoperative; Postop, postoperative; Cr, creatinine; NLR, neutrophil-to-lymphocyte ratio; PLR, platelet-to-lymphocyte ratio; SII, systemic immune-inflammation index; MLR, monocyte-to-lymphocyte ratio; ICU, intensive care unit.

**Table 6:** Clinical reference model and incremental contribution of preoperative inflammatory indices for 30-day mortality.

**Panel A. Clinical Reference Model**					
**Variable**	**Adjusted OR (95% CI)**			***p* Value**	
Age	0.999 (0.964–1.036)			0.972	
Preoperative creatinine	2.58 (1.14–5.85)			**0.023**	
Cross-clamp time (min)	1.023 (1.010–1.036)			**<0.001**	
Total circulatory arrest time (min)	1.522 (1.210–1.915)			**<0.001**	
**Panel B. Incremental Contribution of Each Preoperative Inflammatory Index**					
**Added Biomarker**	**Adjusted OR (95% CI)**	***p* Value**	**Model AUC**	**ΔAUC**	**LR Test *p***
NLR	1.060 (0.987–1.138)	0.108	0.851	0.011	0.112
PLR	1.004 (1.002–1.007)	<0.001	0.883	0.043	<0.001
SII	1.00037 (1.00012–1.00062)	0.004	0.872	0.032	0.002
MLR	1.139 (1.059–1.225)	<0.001	0.859	0.019	<0.001

Reference model AUC = 0.840. Each inflammatory index was entered separately into the clinical reference model because of collinearity among the indices. All multivariable models included 506 patients with 32 30-day mortality events. ΔAUC represents the change from the clinical reference model (AUC = 0.840). LR, likelihood-ratio test; NLR, neutrophil-to-lymphocyte ratio; PLR, platelet-to-lymphocyte ratio; SII, systemic immune-inflammation index; MLR, monocyte-to-lymphocyte ratio.

## Data Availability

The datasets generated and analyzed during the current study are not publicly available due to institutional privacy and ethical restrictions but are available from the corresponding author on reasonable request.

## Abbreviations

ROC, receiver operating characteristic; NLR, neutrophil-to-lymphocyte ratio; PLR, platelet-to-lymphocyte ratio; SII, systemic immune-inflammation index; MLR, monocyte-to-lymphocyte ratio.
